# Perivascular epithelioid cell neoplasm of the urinary bladder in an adolescent: a case report and review of the literature

**DOI:** 10.1186/1746-1596-7-183

**Published:** 2012-12-31

**Authors:** Lijuan Yin, Hong Bu, Min Chen, Jianqun Yu, Hua Zhuang, Jie Chen, Hongying Zhang

**Affiliations:** 1 Department of Pathology, West China Hospital, Sichuan University, Guoxuexiang 37, 610041, Chengdu, Sichuan, China; 2 Department of Radiology, West China Hospital, Sichuan University, 610041, Chengdu, Sichuan, China; 3 Department of Ultrasound, West China Hospital, Sichuan University, 610041, Chengdu, Sichuan, China

**Keywords:** Perivascular epithelioid cell neoplasms, Urinary bladder, Adolescent

## Abstract

**Abstract:**

Perivascular epithelioid cell neoplasms (PEComas) of the urinary bladder are extremely rare and the published cases were comprised predominantly of middle-aged patients. Herein, the authors present the first urinary bladder PEComa occurring in an adolescent. This 16-year-old Chinese girl present with a 3-year history of abdominal discomfort and a solid mass was documented in the urinary bladder by ultrasonography. Two years later, at the age of 18, the patient underwent transurethral resection of the bladder tumor. Microscopically, the tumor was composed of spindled cells mixed with epithelioid cells. Immunohistochemically, the tumor were strongly positive for HMB45, smooth muscle actin, muscle-specific actin, and H-caldesmon. Fluorescence in situ hybridization analysis revealed no evidence of *EWSR1* gene rearrangement. The patient had been in a good status without evidence of recurrence 13 months after surgery. Urinary bladder PEComa is an extremely rare neoplasm and seems occur predominantly in middle-aged patients. However, this peculiar lesion can develop in pediatric population and therefore it should be rigorously distinguished from their mimickers.

**Virtual slides:**

The virtual slide(s) for this article can be found here: http://www.diagnosticpathology.diagnomx.eu/vs/1870004378817301

## Background

Perivascular epithelioid cell neoplasms (PEComas) are defined by the World Health Organization as “mesenchymal tumors composed of histologically and immunohistochemically distinctive perivascular epithelioid cells” [1]. The PEComa family of tumors includes angiomyolipoma (AML), clear cell sugar tumor of the lung (CCST), lymphangioleiomyomatosis (LAM), and rare tumors in other locations [2]. To date, non-AML/non-LAM/non-CCST PEComas have progressively been documented in a variety of anatomical sites, such as visceral organs, soft tissue and bone [3-10]. However, only 12 cases of PEComas of the urinary bladder have been documented in the English-language literature worldwide [11-20]. Herein, we present a urinary bladder lesion occurring in a Chinese adolescent. To the best of our knowledge, the current case is the first published example of bladder PEComa occurring in pediatric population.

## Case presentation

**Figure 1:**
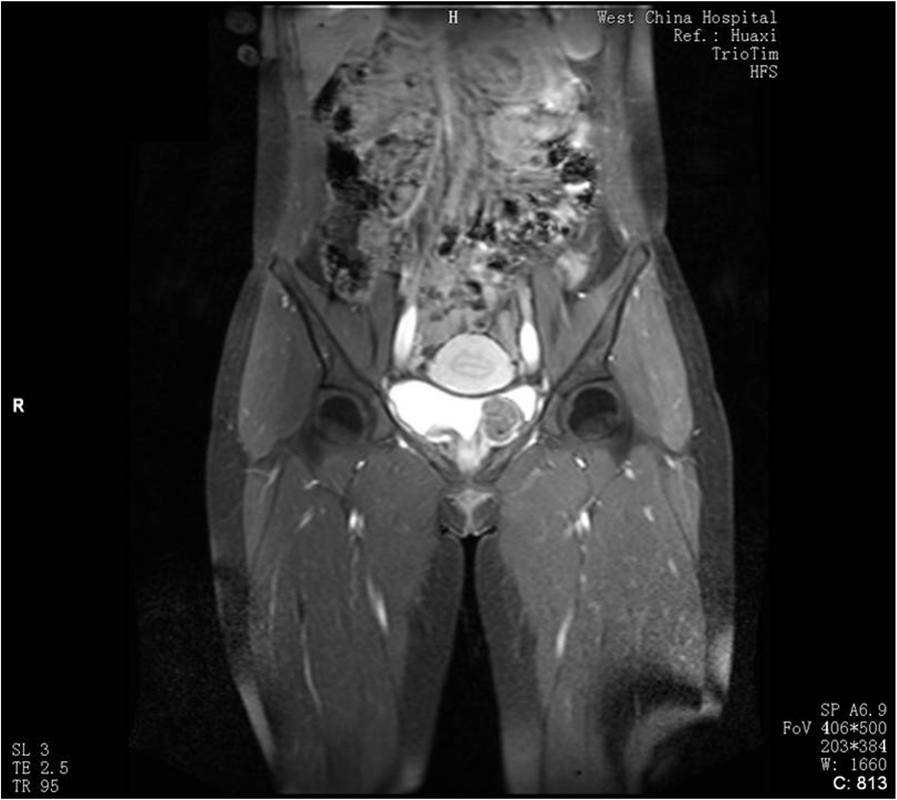
Gd-enhanced coronal T1-weighted MRI showing significantly inhomogeneously enhanced well-defined mass with a wide base.

A 16-year-old girl was referred to a peripheral hospital with a 3-year history of vague abdominal discomfort. A 2.0-cm solid mass was identified in the urinary bladder by ultrasonography. However, her parents preferred to undergo regular follow-up examinations instead of mass resection despite a recommendation. Two years later, at the age of 18, the girl was admitted to our hospital with 1-month history of frequent micturition without hematuria or dysuria. Her family history was unremarkable, and no stigmata of tuberous sclerosis were detected. Ultrasound and computed tomography (CT) scan demonstrated a solid mass arising from the left posterior wall of the bladder. Subsequent magnetic resonance imaging (MRI) of the pelvis revealed a 2.5 × 2.2 cm^2^ sharply circumscribed soft tissue mass, with a wide base. The tumor showed homogeneous intermediate signal intensity on the T1-weighted images, slightly heterogeneous hyperintense signals on the T2-weghted images, and significant inhomogeneous enhanced signals on the T1-weighted images (Figure 1). Cystoscopy demonstrated a yellowish solid mass approximately 3 cm in diameter located in the bladder wall, 2 cm below the left ureteral orifice, which was partially protruding into the bladder lumen. Subsequently, the patient underwent transurethral resection of the bladder tumor (TURBT). Both the intraoperative impression and a postoperative ultrasound confirmed that gross-total tumor resection had been achieved.

**Figure 2:**
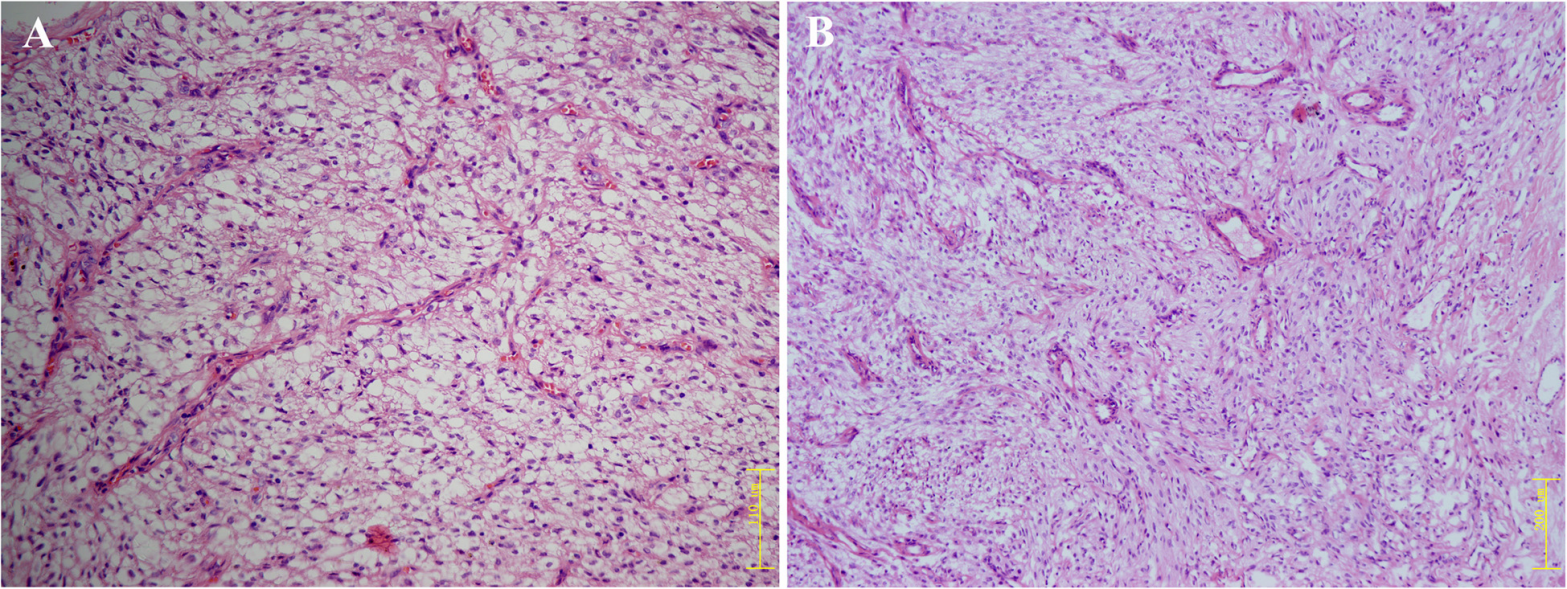
**Histologic features (Hematoxylin and eosin staining). A**: The tumor consisting of spindled cells mixed with epithelioid cells, exhibiting clear to lightly eosinophilic cytoplasm. (Original magnification: 200×). **B**: The neoplastic cells arranged around an elaborate capillary-sized vascular network. (Original magnification: 100×).

**Figure 3:**
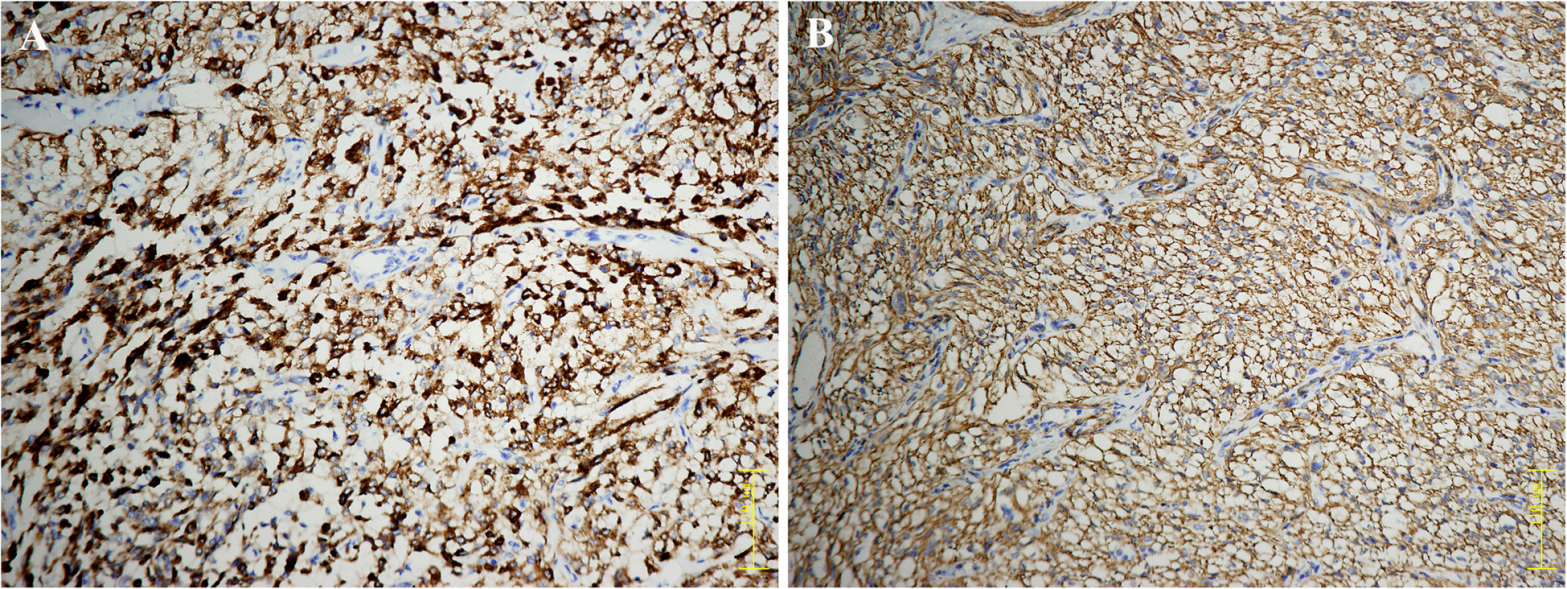
**Immunohistochemical analysis. A**: Positive staining for HMB45. **B**: Positive staining for smooth muscle actin (SMA). (Original magnification: 200×).

A fragmented specimen, 3 cm in aggregate, was subjected to pathological examination. Histologically, the tumor was composed of spindled cells mixed with epithelioid cells arranged in fascicles or nests, with clear to lightly eosinophilic cytoplasm (Figure 2A). Notably, an elaborate capillary-sized vascular network was interspersed between the cellular fascicles or nests (Figure 2B). The cellularity was low to moderate. The tumor cells were generally bland and uniform without prominent nuclear atypia or increased mitotic activity (0/50 HPF). The nuclei were round to oval in shape with inconspicuous nucleoli. Neither necrosis nor vascular invasion was observed. Further immunohistochemical staining revealed that the neoplastic cells were strongly positive for HMB45 (Figure 3A), smooth muscle actin (SMA) (Figure 3B), muscle-specific actin (MSA), and H-caldesmon. The neoplastic cells were negative for the remaining antibodies, including Melan-A, desmin, ALK-1, myogenin, S-100 protein, pan-cytokeratin (AE1/AE3), TFE3, and EMA. Evaluation of proliferative activity with MIB-1 LI was lower than 2% of the neoplastic cells. Additionally, fluorescence in situ hybridization analysis revealed no evidence of 22q12 (*EWSR1* gene) translocation, excluding the possibility of clear cell sarcoma of soft tissue (CCSST). The histopathological features, together with immunohistochemical pattern and genetic studies, indicated that the tumor was a PEComa.

The patient’s symptoms were almost relieved completely and she was discharged on the 5th postoperative day. Six months later, a follow-up CT scan and ultrasound were normal. At the most recent follow-up 13 months following the surgery, the patient had been in a good status without evidence of recurrence.

## Discussion

PEComa belongs to a peculiar neoplasm and accounts for an extremely smaller percentage of urinary bladder mesenchymal lesions. A search of the English literature indicated that only 12 previous cases of PEComas of the urinary bladder have been reported thus far [11-20], whereas two cases mentioned in Chinese-language literatures with English-language abstract were excluded because of the unavailable detail information [21,22]. The clinicopathologic features of all published cases of this kind of lesion are summarized in Table 1. Of the total of 13 cases, including the one in this context, the female-to-male ratio was 6:7. The age of the patients ranged from 16 to 48 years (median: 36 years; average: 32.8 years). The published cases seem to occur predominantly in middle-aged patients. However, the current patient is the youngest one and represents the first case involving the pediatric patient although less than 40 pediatric PEComas have been reported in other locations [23]. It is also noteworthy to mention that this patient present with a 3-year history of abdominal discomfort, implying that this lesion already existed during childhood. Thus, urinary bladder PEComas could develop very early in life. PEComas can arise from any region of the bladder wall without overt anatomical preference. All of the 13 bladder PEComas were solitary lesions, although multifocal PEComas haven been deocumented in other locations [24]. The tumor size of the 13 cases ranged from 2.5 to 9.2 cm (median: 3.7 cm; average: 4.3 cm). The clinical presentation comprised non-specific urinary symptoms, such as hematuria, odynuria, and vague abdominal pain. None of the patients was associated with the tuberous sclerosis complex.

**Table 1:** Reported cases of the urinary bladder PEComas in the English-Language Literature and their clinicopathologic features

**Case**	**Authors/Year/**	**Age(y) /Sex**	**Symptoms**	**Site/Size (cm)**	**Border**	**Nuclear grade and cellularity**	**MF/ 50 HPF**	**Necrosis**	**Vascular invasion**	**Immunohistochemistry**	**Treatment**	**Outcomes/follow-up (mo)**
1	Pan 2003 [11]	33/F	Symptomless	Left lateral inferior /4.0	W	U	0	No	U	HMB45 (+), SMA (+), vimentin (−), desmin (−),S-100 protein (−), Melan-A (−), pan-cytokeratin (KL-1) (−), ER (−), PR (−)	Partial cystectomy	ANEDx72
2	Kalyanasundaram 2005 [12]	19/F	Hematuria	Left lateral /3.0	I	High	Few	U	U	HMB45 (+), cytokeratin (−), S-100 (−), synaptophysin (−), vimentin (−), desmin (−), chromogranin A (−)	TURBT	U
3	Parfitt 2006 [13]	48/M	Lower abdominal pain and dysuria	Posterior mid-wall /3.0	I	High	0	Inconspicuous	U	HMB45 (+), Melan-A (+), SMA (+), desmin (+), c-kit (+), S-100 protein (−), pan-cytokeratin (AE1/AE3) (−), vimentin (−), MSA (−), myoglobin (−), CD31 (−), CD34 (−), WT-1 (−)	Partial cystectomy and partial small bowel resection with adjuvant INF-a immunotherapy	ANEDx48
4	Weinreb 2007 [14]	39/M	U	Dome/urachal remnant cyst /5.0	I, focally	High, focal	Isolated	Yes, focal	No	HMB45 (+), SMA (+), MSA (+), desmin (+), Melan-A (+), S-100 protein (+), MiTF (+), cyclin D1 (+)	Partial cystectomy	ANEDx8
5	Pianezza 2008 [15]	24/F	Chronic pelvic pain	Posterior/3.4	I, focally	Low to moderate	1	No	U	HMB45 (+), S-100 protein (+), H-caldesmon (+), actin (+), Melan-A (−), CD34 (−), A103 (−), pankeratin (−), calretinin (−), CD99 (−), ALK-1 (−), c-kit (−), desmin (−)	Partial cystectomy	ANEDx3
6	Sukov 2009 [16]	36/M	Hematuria	Anterior /4.8	I	Low to moderate	0	Yes, focal	U	HMB45 (+), SMA (+), tyrosinase (+), desmin (−), Melan-A (−)	Partial cystectomy	ANEDx10
7		37/M	Hematuria	Dome /U	W	Low to moderate	0	No	U	HMB45 (+), pan-cytokeratin (−), vimentin (−), CD56 (−), chromogranin A (−), Melan-A (−), S-100 protein (−), synaptophysin (−), SMA (−), high-molecular-weight cytokeratin (−), inhibin (−)	TURBT	ANEDx21
8		26/F	U	Anterior /5.0	W	Low	0	Yes (related to embolization)	U	HMB45 (+), SMA (+), vimentin (+), Melan-A (−), tyrosinase (+), S-100 protein (−), c-kit (−), MSA (−), cytokeratin (−)	Embolization and partial cystectomy	U
9	Huang 2011 [17]	23/M	Frequent micturition and odynuria	Left lateral /9.2	W	Low	0	U	U	HMB45 (+), vimentin (+), SMA (+), S-100 protein (−), Melan-A (−), synaptophysin (−), c-kit (−), CD34 (−), chromogranin A (−), cytokeratin 8 (−)	Tumorectomy and partial cystectomy	U
10	Chan 2011 [18]	42/M	Vague urethral pain	Right lateral /6.0	W	Low to moderate?	<1	No	U	HMB45 (+), S-100 protein (+), MiTF (+), SMA (+), calponin (+), vimentin (+), desmin (−), myogenin (−),TFE3 (−), pan-cytokeratin (AE1/AE3) (−), CD34 (−), ALK-1 (−), synaptophysin (−), c-kit (−), Ki67 (+, 1%)	TURBT and then partial cystectomy	U
11	Kyrou 2012 [19]	44/F	Dysmenorrhea	Left posterior /2.7	U	U	0	No	U	HMB45(+), S-100 protein (+), EMA (−), cytokeratin (−), desmin(−)	Partial colpectomy and cystectomy/ right ovariectomy/ pelvic lymphadenectomy	ANEDx30
12	Shringarpure 2012 [20]	39/M	Painless hematuria	Left vesicoureteric junction/3	U	U	U	U	U	HMB45 (+), Melan-A (+), SMA (+), vimentin (+), MSA (+), S-100 protein (+), cytokeratin (−), desmin(−)	TURBT	ANEDx3
13	Present case	16/F	Abdominal discomfort and micturition	Posterior/2.5	W	Low	0	No	No	HMB45 (+), SMA (+), MSA (+), desmin (−), H-caldesmon (+), Melan-A (−), ALK-1 (−), myogenin (−), EMA (−), pan-cytokeratin (AE1/AE3) (−), S-100 protein (−), Ki-67 (+, 2%)	TURBT	ANEDx13

W indicates well-circumscribed; *I*, infiltrative; *MF*, mitotic figures; *TURBT*, transurethral resection of bladder tumor; *ANED*, alive, not evidence of disease; *U*, unknown.

Microscopically, all of the 13 cases exhibited classic features of PEComa, with admixture of epithelioid and spindled cells arranged radially around blood vessels. Five of 11 cases exhibited infiltrative growth pattern. The mitotic activity of all of the cases was no more than 1/50 HPF. Necrosis was found in 3 of 10 patients, although the necrosis in 1 of the 3 cases might be attributed to prior embolization [16]. Vascular invasion information was limited. Immunohistochemically, most cases exhibited co-expression of melanotic and muscle markers, consistent with immunophenotype of classic PEComa. Notably, HMB45 was the most sensitive marker and all of the 13 cases (100%) showed strong positivity for this reagent. Diffuse immunoreactivity for SMA was observed in 91% (10/11) of cases. In addition, neoplastic cells also showed variable staining for other markers. For instance, Melan-A, desmin, and S-100 protein expression was identified in 30% (3/10), 30% (3/10), 42% (5/12) of lesions, respectively.

The urinary bladder is an extremely rare location for PEComa, especially for pediatric population. The differential diagnosis of this peculiar lesion is broad and sometimes might be very challenging especially in small biopsy samples. This unique lesion must be differentiated from melanotic tumors and other more common types of pediatric bladder mesenchymal lesions, such as smooth muscle tumors, rhabdomyosarcoma, and pseudosarcomatous myofibroblastic proliferation.

This unique lesion can be confused with CCSST and melanoma because of the strong positivity for melanotic markers, especially when neoplastic cells express S-100 protein. However, melanotic tumors can be distinguished from PEComa in several respects. First, cells of melanotic neoplasms are characterized by large and conspicuous nucleoli. Second, strong S-100 protein expression is usually detected in most melanotic tumors but only in a minority of PEComas [19]. Third, the majority of PEComas also express muscle markers, whereas most melanotic tumors are negative for actin. Fourth, and most importantly, identification of t(12;22)(q13;q12)(*EWSR1-ATF1*) or t(2;22)(q34;q12)(*EWSR1/CREB1*) fusion can be invaluable in distinguishing CCSST from PEComa [25].

Smooth muscle tumors can mimic PEComa, as both tumor types express muscle markers. Smooth muscle tumors usually arrange in fascicles, demonstrating ‘cigar-shaped’ nuclei and more eosinophilic cytoplasm. Immunohistochemically, the majority of smooth muscle tumors express strong positivity for desmin, whereas PEComas are usually desmin-negative or express desmin only focally. It is noteworthy to mention that very rare true smooth muscle tumors can express HMB45 [26]; however, the staining is focally and usually not strong, while most of PEComas usually show diffuse and strong HMB45 expression.

Both rhabdomyosarcoma and pseudosarcomatous myofibroblastic proliferation are more common in this site, especially for very young patients. Additionally, these two lesions express muscle markers. However, detailed morphologic inspection combined with negativity for melanotic markers in these two lesions can be very valuable in distinguishing them from PEComas [27-29].

Lastly, pediatric PEComas of the urinary system sometimes may be confused with renal carcinoma associated with Xp11.2 translocations/*TFE3* gene fusions, which usually affect children and young adults [30-32]. Of note, a small minority of this peculiar renal carcinoma may demonstrate immunoreactivity for HMB45 and is usually negative for epithelial markers [30,32]. However, careful morphologic inspection, in conjunction with the immunostaining pattern and even genetic studies can be useful in this differential diagnosis.

In 2005, a criterion for the classification of PEComas as “benign”, “uncertain malignant potential” and “malignant” was proposed by Folpe et al. [27]. Most PEComas behave in a benign fashion and rare clinically malignant PEComas with distant metastasis have been described [7-10,33]. Following this classification system suggested by Folpe et al., at least 4 of the cases (cases 2, 3, 4, and 6) could be classified as malignant lesions. However, based on the follow-up information available in 9 of the cases (including 3 of the malignant lesions), there have been no reports of recurrence or metastasis to date, implying that PEComas of urinary bladder might be indolent. However, the following factors need to be considered prior to drawing such a conclusion. First, the number of cases was limited, and the clinical follow-up period (average: 22.8 months; range: 3–72 months) of >12 months was available only in 5 cases (average: 36.8 months; range: 13–72 months). Second, whether the histological grade of urinary bladder PEComas exhibits a linear association with their clinical behavior remains unknown. Generally speaking, the behavior of urinary bladder PEComas seems to be unpredictable, indicating the necessity for the further investigation of more cases with long-term follow-up.

Currently, the optimal treatment for PEComas is not known. Among the 13 cases, 9 patients underwent partial cystectomy and 4 of them received TURBT. Complete excision seems to be curative and might be necessary to avoid progression. Notably, 1 patient was treated by postoperative adjuvant INF-α immunotherapy [13]. However, further investigation is warranted to evaluate the efficacy of adjuvant postoperative therapy in these unusual lesions.

## Conclusion

Urinary bladder PEComa is an extremely rare neoplasm and usually occur in middle-aged patients. The present case represents the first one occurring in an adolescent, indicating that this peculiar lesion can affect pediatric population. Therefore, pediatric bladder PEComa should be rigorously distinguished from other more common types of bladder lesions. Bladder PEComas exhibited classic features of PEComa. All of the lesions seem to behave in an indolent fashion although some tumors revealed malignant morphology. Nonetheless,, the behavior of PEComa in this site remains unpredictable. Further studies, including additional large numbers of cases and longer-term follow-up periods are necessary. Additionally, cytogenetic or molecular biological investigation is also warranted to better delineate this peculiar lesion.

## Consent

Written informed consent was obtained from the patient for publication of this case report and accompanying images. A copy of the written consent is available for review by the Editor-in-Chief of this journal.

## Abbreviations

PEComa: Perivascular epithelioid cell neoplasm; AML: Angiomyolipoma; LAM: Lymphangioleiomyomatosis; CCST: Clear cell sugar tumor of the lung; CCSST: Clear cell sarcoma of soft tissue.

## Competing interests

The authors declare that they have no competing interests.

## Authors’ contributions

LY made contributions to acquisition of clinical data, pathological analysis and manuscript writing. HB drafted the manuscript. MC and JC carried out the genetic studies. JY participated in the radiological analysis; HZ (Hua Zhuang) participated in the ultrasonography diagnosis. HZ (Hongying Zhang) conceived of the study, and participated in its design and coordination and helped to draft and edit the manuscript. All authors read and approved the final manuscript.

## Funding

This study was supported by National Natural Science Foundation of China (no. 30971148 and no. 81272944).
